# Supplementary material for: Influence of the Sputtering Temperature on Reflectivity and Electrical Properties of ITO/AgIn/ITO Composite Films for High-Reflectivity Anodes
Source: Materials (Basel). 2023 Apr 3;16(7):2849. doi: 10.3390/ma16072849 (PMC10095718; doi:10.3390/ma16072849)
Supplement: Supplementary file 1 [file materials-16-02849-s001.zip › materials-2293790-supplementary.pdf]

Influence of the Sputtering Temperature on Reflectivity and Electrical Properties of ITO/AgIn/ITO Composite Films for High-Reflectivity Anodes

Xianqi Wang [1], Hongda Zhao [2], Bo Yang [1,*], Song Li [1,*], Zongbin Li [1], Haile Yan [1], Yudong Zhang[3], Claude Esling[3], Xiang Zhao [1] and Liang Zuo [1]

[1] Key Laboratory for Anisotropy and Texture of Materials, School of Material Science and Engineering, Northeastern University, Shenyang 110819, China

[2] Shenyang Dong Chuang Precious Metals Material Co., Ltd. Shenyang 110000, China

[3] Laboratoire d'Étude des Microstructures et de Mécanique des Matériaux, (LEM3), CNRS, UMR 7239, University of Lorraine, 57045 Metz, France

*Corresponding author: yangb@atm.neu.edu.cn (B.Y.); lis@atm.neu.edu.cn (S. L.)

Supplementary S1. Influence of sputtering temperature on the structure and properties of AgIn/Glass

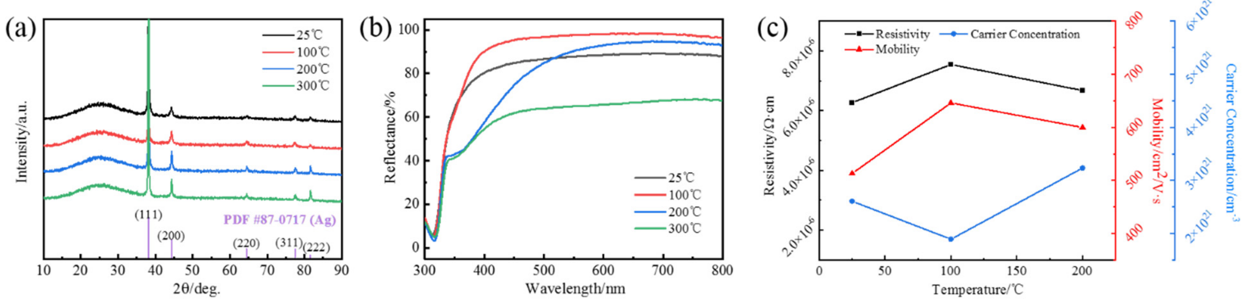

Figure S1. (a) XRD pattern of the AgIn/Glass films as a function of sputtering temperature. (b) Reflectance spectra of the AgIn/Glass films as a function of sputtering temperature. (c) Hall measurement of the AgIn/Glass films as a function of sputtering temperature.

Supplementary S2. Influence of sputtering temperature on the structure and properties of ITO/Glass

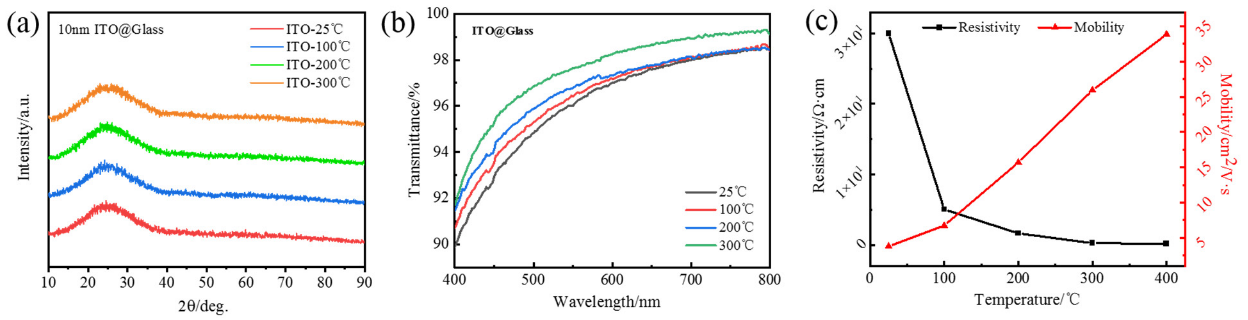

Figure S2. (a) XRD pattern of the ITO/Glass films as a function of sputtering temperature. (b)Transmittance spectra of the ITO/Glass films as a function of sputtering temperature. (c) Hall measurement of the ITO/Glass films as a function of sputtering temperature.

Supplementary S3. Comparison of SEM images of ITO/AgIn/ITO films and AgIn films at different sputtering temperatures.

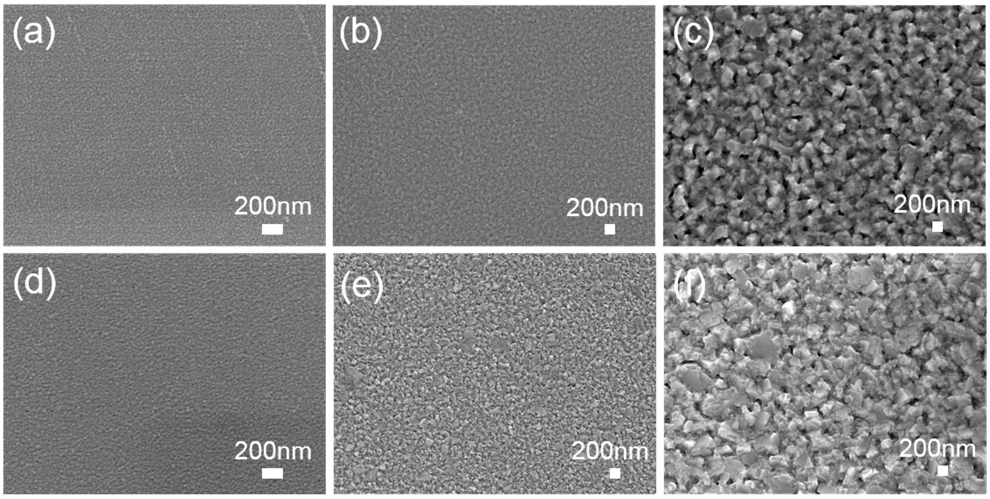

Figure S3. Comparison of SEM images of ITO/AgIn/ITO films and AgIn films at different sputtering temperatures. ITO/AgIn/ITO films: (a) 25°C, (b) 100°C and (c) 200°C; AgIn films: (d) 25°C, (e) 100°C and (f) 200°C.
